# Muscle dysfunction associated with adjuvant-induced arthritis is prevented by antioxidant treatment

**DOI:** 10.1186/s13395-015-0045-7

**Published:** 2015-07-09

**Authors:** Takashi Yamada, Masami Abe, Jaesik Lee, Daisuke Tatebayashi, Koichi Himori, Keita Kanzaki, Masanobu Wada, Joseph D. Bruton, Håkan Westerblad, Johanna T. Lanner

**Affiliations:** Graduate School of Health Sciences, Sapporo Medical University, South 1 West 17, Chuo-ku, 060-8556, Sapporo Japan; Faculty of Food Culture, Kurashiki Sakuyo University, 3515 Nagao-Tamashima, Kurashiki, Japan; Graduate School of Integrated Arts and Sciences, Hiroshima University, 1-7-1, Higashi, Hiroshima Japan; Department of Physiology and Pharmacology, Karolinska Institutet, SE-17177 Stockholm, Sweden

**Keywords:** Rheumatoid arthritis, Muscle weakness, Aggregation, Antioxidant

## Abstract

**Background:**

In addition to the primary symptoms arising from inflamed joints, muscle weakness is prominent and frequent in patients with rheumatoid arthritis (RA). Here, we investigated the mechanisms of arthritis-induced muscle dysfunction in rats with adjuvant-induced arthritis (AIA).

**Methods:**

AIA was induced in the knees of rats by injection of complete Freund’s adjuvant and was allowed to develop for 21 days. Muscle contractile function was assessed in isolated extensor digitorum longus (EDL) muscles. To assess mechanisms underlying contractile dysfunction, we measured redox modifications, redox enzymes and inflammatory mediators, and activity of actomyosin ATPase and sarcoplasmic reticulum (SR) Ca^2+^-ATPase.

**Results:**

EDL muscles from AIA rats showed decreased tetanic force per cross-sectional area and slowed twitch contraction and relaxation. These contractile dysfunctions in AIA muscles were accompanied by marked decreases in actomyosin ATPase and SR Ca^2+^-ATPase activities. Actin aggregates were observed in AIA muscles, and these contained high levels of 3-nitrotyrosine and malondialdehyde-protein adducts. AIA muscles showed increased protein expression of NADPH oxidase 2/gp91^phox^, neuronal nitric oxide synthase, tumor necrosis factor α (TNF-α), and high-mobility group box 1 (HMGB1). Treatment of AIA rats with EUK-134 (3 mg/kg/day), a superoxide dismutase/catalase mimetic, prevented both the decrease in tetanic force and the formation of actin aggregates in EDL muscles without having any beneficial effect on the arthritis development.

**Conclusions:**

Antioxidant treatment prevented the development of oxidant-induced actin aggregates and contractile dysfunction in the skeletal muscle of AIA rats. This implies that antioxidant treatment can be used to effectively counteract muscle weakness in inflammatory conditions.

## Background

Patients with rheumatoid arthritis (RA) show significant muscle weakness, and this has a major impact on the disability in these patients [1]. Loss of muscle strength in RA patients was traditionally attributed to a disuse atrophy caused by joint deformity and pain [2]. However, functional studies show that decreased specific force (i.e., force per cross-sectional area) makes an equally important contribution to the overall weakness in RA patients [3, 4]. Moreover, we reported a marked depression in maximal specific force in both fast-twitch extensor digitorum longus (EDL) and flexor digitorum brevis and slow-twitch soleus muscle from collagen-induced arthritis (CIA) mice, a widely used animal model for RA [5, 6]. It is worth noting that intrinsic contractile properties of fast-twitch muscle are little affected in animal models of disuse [7, 8].

The peroxynitrite anion (ONOO^−^) is an oxidant formed when nitric oxide (NO) reacts with superoxide. We previously showed that the depressed myofibrillar force production in CIA muscles was accompanied by an increased 3-nitrotyrosine (3-NT) content [5, 6], which is regarded as a footprint of increased ONOO^−^ production [9]. In addition, ONOO^−^ exposure depressed Ca^2+^-activated force production in skinned muscle fibers from rat [10]. Thus, impaired myofibrillar function mediated by ONOO^−^ is likely to be important in arthritis-induced muscle dysfunction. Furthermore, Tiago et al. [11] showed that actin is more sensitive to ONOO^−^ than other proteins in the contractile machinery (i.e., myofibrillar proteins including myosin) and that ONOO^−^-induced oxidation of actin inhibits the ability of actin to stimulate actomyosin ATPase activity.

EUK is a novel cell-permeable manganese-salen (Mn-Salen) compound, which mimics the catalytic function of superoxide dismutase (SOD) and catalase. EUK has been shown to eliminate not only superoxide and hydrogen peroxide [12] but also NO-derived radicals, including ONOO^−^ via additional oxidation of EUK [13]. Several studies have shown that administration of EUK protects against oxidative stress-induced muscle weakness and wasting in various conditions [14–16]. Specifically, EUK-134 prevented reductions in specific force generation in the diaphragm muscle of mdx mice [15].

Here, we examined mechanisms underlying arthritis-induced muscle weakness, focusing on the role of redox stress on contractile properties in fast-twitch EDL muscles. The major results show oxidant-induced actin aggregates and contractile dysfunction in muscles of rats with adjuvant-induced arthritis (AIA), and these deleterious changes were prevented when rats were treated with the antioxidant EUK-134.

## Methods

### Ethical approval

All experimental procedures were approved by the Committee on Animal Experiments of Sapporo Medical University. Animal care was in accordance with the institutional guidelines.

### Induction of adjuvant-induced arthritis

Male Lewis rats (8-week old, *n* = 28) were supplied by Sankyo Labo Service (Sapporo, Japan). Rats were given food and water ad libitum and housed in an environmentally controlled room (24 ± 2 °C) with a 12-h light-dark cycle. AIA was induced in the knees by an intraarticular injection of 0.2 ml of cocktail containing Freund’s incomplete adjuvant (DIFCO) and 2 mg *Mycobacterium butyricum* (DIFCO) under sodium pentobarbital (40 mg/kg) anesthesia. Some rats were treated with the superoxide dismutase/catalase mimetic EUK-134 (Calbiochem). EUK-134 was dissolved in phosphate-buffered saline (PBS) at 2 mg/ml, and rats were injected intraperitoneally at a dose of 3 mg/kg/day starting 1 day after induction of AIA. PBS was injected as a control.

Three weeks after induction of AIA, rats were killed by an overdose of sodium pentobarbital (120 mg/kg). The maximum diameters of the knee joint were measured with a Mitutoyo caliper. The fast-twitch extensor digitorum longus (EDL) muscles were excised; in some experiments, we also excised slow-twitch soleus muscles.

### Measurement of isometric contractile force

The force-frequency relationship was established in intact EDL and soleus muscles, as described previously [5]. For twitch contractions, the peak rate of tension development (+dP/d*t*, maximal positive slope of the force record determined over a 10-ms moving window) and the peak rate of tension decline (−dP/d*t*, maximum negative slope of the force record measured over a 10-ms moving window) were determined. Absolute force was normalized to cross-sectional area, calculated as muscle weight divided by the length giving maximum tetanic force and density (1056 kg m^−3^).

### Myosin heavy chain and actin content in myofibrillar proteins

Extraction of myofibrils was performed as previously described [17]. Myofibrillar proteins were separated by a 10 % SDS-PAGE gel. Aliquots of myofibrillar extracts containing 5 μg of protein were subjected to electrophoresis and stained with Coomassie brilliant blue. Images of gels were acquired using the GelDoc imaging system (Bio-Rad). The relative content of myosin heavy chain (MyHC) or actin in total myofibrillar proteins was evaluated densitometrically using ImageJ.

### MyHC isoforms separation

Aliquots of the extracts containing 5 μg myofibrillar protein were applied for MyHC electrophoresis as previously described in detail [18]. Using a 6 % polyacrylamide slab gel, electrophoresis was run at 4 °C for 48 h at 160 V and stained with Coomassie brilliant blue. Images of gels were densitometrically evaluated with ImageJ.

### Actomyosin ATPase activity

Actomyosin ATPase activity was spectrophotometrically determined in myofibrillar extracts at 37 °C using a previously described assay [17]. Western blotting confirmed that the myofibrillar extracts contained no sarcoplasmic reticulum (SR) Ca^2+^-ATPase (SERCA) (data not shown). An aliquot of myofibrillar extracts was added to the reaction mixture (mM): KCl, 30; Tris, 30; sodium azide, 2; MgSO_4_, 1; EGTA, 1; CaCl_2_, 1.1; NADH, 0.4; phosphoenolpyruvate, 10; 18 U/ml pyruvate kinase; and 18 U/ml lactate dehydrogenase. The reaction was then started by adding Na-ATP to give a final concentration of 1 mM. The oxidation of NADH was monitored in a spectrophotometer (340 nm) for 3 min. Actomyosin ATPase activity is expressed as millimole/minute/gram myofibrillar proteins.

### Sarcoplasmic reticulum Ca^2+^-ATPase activity

Muscle pieces were homogenized in ice-cold homogenizing buffer (9 μl/mg wet wt) consisting of (mM): sucrose, 300; Mops/KOH, 20; and one tablet of protease inhibitor cocktail (Roche) per 50 ml (pH 7.4). The lysate was then centrifuged at 5000*g* for 10 min. The protein content of the supernatant was determined using Bradford assay [19] and stored at −80 °C. SERCA activity was measured as described previously [20]. Briefly, aliquots of the supernatant (20 μl) were applied to the assay mixture (mM): Hepes, 20; EGTA, 1; KCl, 200; MgCl_2_, 15; CaCl_2_, 0.8; NaN_3_, 10; NADH, 0.4; phosphoenolpyruvate, 10; 12.1 U/ml pyruvate kinase; 20.2 U/ml lactate dehydrogenase; and 1 μg/ml Ca^2+^ ionophore A23187 (Sigma) (pH 7.1). The reaction was initiated by adding Mg-ATP at a final concentration of 4 mM. Finally, the CaCl_2_ concentration was increased to 20 mM to selectively inhibit SERCA activity. The remaining activity was defined as the background ATPase activity. The activity of SERCA was calculated as the difference between the total and the background ATPase activities and expressed as millimole/minute/gram cytoplasmic proteins.

### Immunoblots

Crude muscle homogenates were separated by electrophoresis and transferred onto membranes. Membranes were incubated with primary antibody (anti-ryanodine receptor 1 (RyR1), MA3-925, Thermo; anti-dihydropyridine receptor (DHPR) α2 subunit, ab2864, Abcam; anti-SERCA1, MA3-911, Thermo; anti-SERCA2, ab3625, Abcam; anti-actin, A4700, Sigma; anti-troponin I, MAB1691, Millipore; anti-3-NT, ab52309, Abcam; anti-malondialdehyde (MDA), MD20A-R1a, Academy Bio-Medical; anti-tumor necrosis factor α (TNF-α), 11948, Cell Signaling; anti-high-mobility group box 1 (HMGB1), 326059652, SHINO-TEST; anti-NADPH oxidase 2 catalytic subunit gp91^phox^ (NOX2/gp91^phox^), ab31092, Abcam; anti-neuronal, anti-endothelial, and anti-inducible nitric oxide synthases (nNOS, eNOS, and iNOS, respectively), 610308, 610296, 610328, respectively, BD Biosciences; anti-manganese SOD (SOD2), 06-984, Upstate). Images of the membrane were collected following exposure to chemiluminescence substrate (Millipore) using a charge-coupled device camera attached to ChemiDOC MP (Bio-Rad), and Image Lab Software was used for detection as well as densitometry.

### Statistics

Data are presented as mean ± SEM. Student’s unpaired *t* tests, as well as one-way ANOVA and one-way repeated measures ANOVA, were used to determine statistically significant differences as appropriate. A Bonferroni post hoc test was used when significant differences were determined using ANOVA. A *P* value less than 0.05 was regarded as statistically significant.

## Results

### Knee width, body weights, and muscle weights

All rats exposed to intraarticular knee injection of Freund’s incomplete adjuvant and *Mycobacterium butyricum* developed AIA, and the maximum diameter of the knee joint of AIA rats was significantly increased by 20 % compared to the control rats (11.8 ± 0.3 versus 9.7 ± 0.1 mm (*n* = 6); *P* < 0.01). The body weights of the rats with AIA were significantly lower than those of the control group (271 ± 9 versus 320 ± 6 g (*n* = 6); *P* < 0.01). In addition, absolute EDL muscle weight was ~10 % lower in the rats with AIA than that in controls (115 ± 5 versus 128 ± 3 mg (*n* = 6); *P* < 0.05), but this difference disappeared when muscle weight was normalized to body weight (0.4 ± 0.01 versus 0.4 ± 0.01 mg/g (*n* = 6); *P* > 0.05). For the soleus muscles, both the absolute and the normalized muscle weights were significantly lower in the rats with AIA than in the control rats (75 ± 6 versus 117 ± 2 mg (*n* = 6–7); *P* < 0.01; 0.28 ± 0.01 versus 0.36 ± 0.01 mg/g (n = 6–7); *P* < 0.01).

### AIA causes a decreased actomyosin ATPase and SERCA activity

**Fig. 1:**
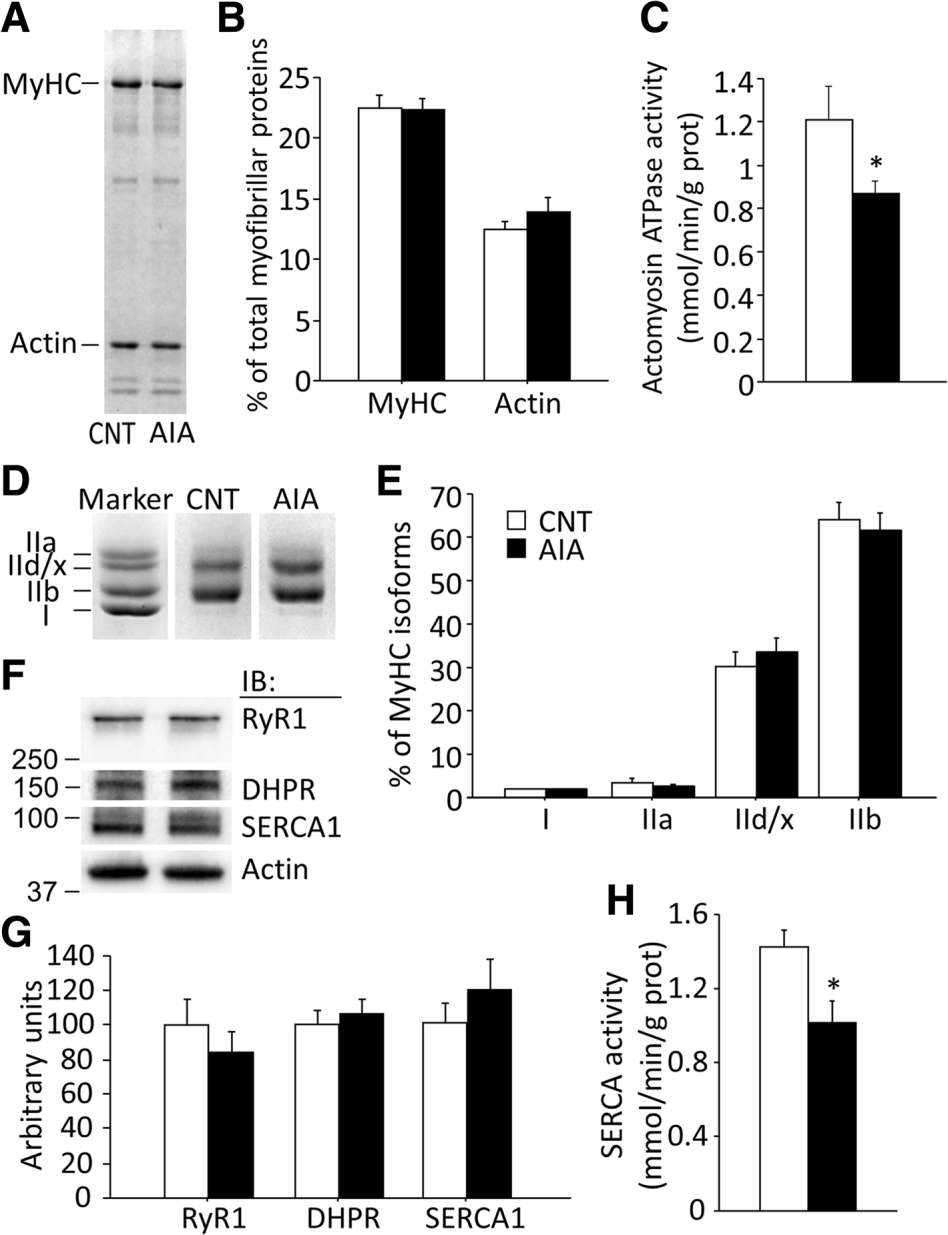
Actomyosin ATPase and sarcoplasmic reticulum (SR) Ca^2+^-ATPase activities are decreased in AIA EDL muscles. Representative Coomassie brilliant blue staining of myofibrillar proteins (**a**), percentage distribution of myosin heavy chain (MyHC) and actin content in total myofibrillar proteins (**b**), and actomyosin ATPase activity (**c**). Electrophoretically separated MyHC isoforms (**d**) and percentage distribution of MyHC isoforms (**e**): I, slow isoform and IIa, IId/x, and IIb, fast isoforms. Representative Western blots of ryanodine receptor (RyR1), dihydropyridine receptor α2 subunit (DHPR), and SR Ca^2+^-ATPase (SERCA) 1 (**f**), the expression levels of these proteins normalized to actin content (**g**), and SERCA activity (**h**). Control (CNT), *white bars*; AIA, *black bars*. Data are presented as mean ± SEM for three to seven muscles in each group. *P < 0.05 versus CNT

There was no difference in the total myofibrillar protein concentration in EDL muscles between the rats with AIA and control rats (130.0 ± 5.7 versus 120.0 ± 13.4 mg/g wet wt (*n* = 7–8); *P* > 0.05). A minor reduction in MyHC, but not actin, protein expression has been observed in CIA soleus muscle [5]. We therefore assessed the expression levels of MyHC and actin. Figure 1a shows a typical expression pattern of these myofibrillar proteins in control and AIA muscles. Neither the MyHC nor actin content was altered in AIA muscles compared with control muscles (Fig. 1b).

In contrast, the actomyosin ATPase activity in muscles of AIA rats was reduced to ~70 % than that in controls (Fig. 1c). Rat EDL muscles are composed mainly of fast-twitch fibers (Fig. 1d). There were no significant differences in the distribution of the MyHC isoforms between the control rats and rats with AIA (Fig. 1e). Thus, the decreased actomyosin ATPase activity observed in our study was not due to changes in the expression of MyHC isoforms.

There were no changes in the expression levels of the Ca^2+^-handling proteins RyR1, DHPR, or SERCA1 (Fig. 1f, g), whereas SERCA2 was not detected in either group. In contrast, SERCA activity was ~30 % lower in AIA than that in control EDL muscles (Fig. 1h). Thus, AIA muscles display impaired intrinsic function of both SERCA and actomyosin, i.e., the two major ATPases in the skeletal muscle.

### Antioxidant does not prevent arthritis but protects against AIA-induced muscle weakness

**Fig. 2:**
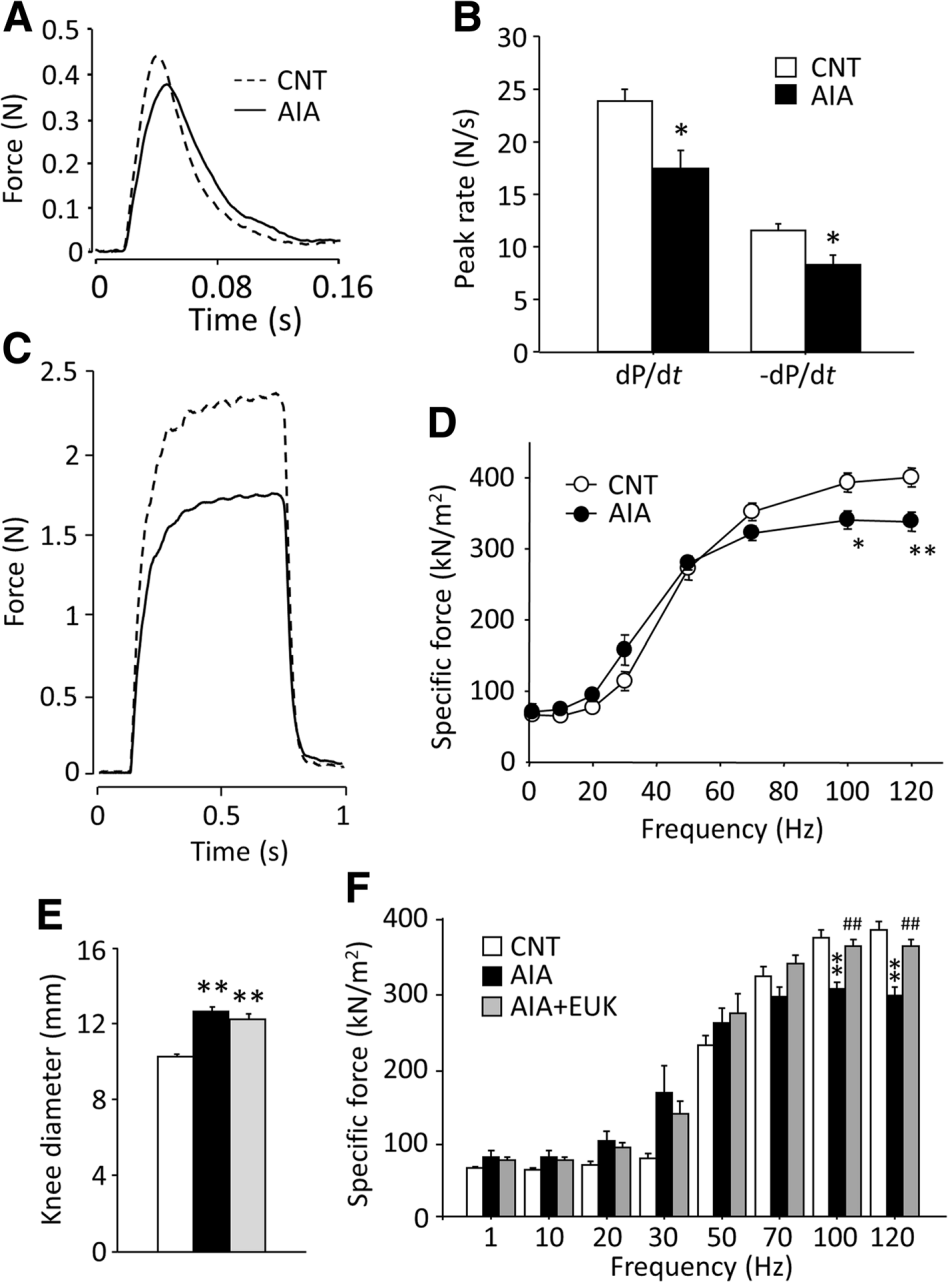
Antioxidant treatment prevents contractile dysfunction in AIA EDL muscles. Representative original records of twitch (**a**) and 100 Hz tetanic (**c**) force from a control (CNT, *dashed line*) and an AIA (*full line*) EDL muscle. The peak rates of twitch force development (dP/d*t*) and relaxation (−dP/d*t*) (**b**) and specific force-frequency relationship (**d**) in CNT and AIA EDL muscles. Maximum knee diameter (**e**) and specific force-frequency relationship (**f**) in EDL muscles from CNT and AIA rats with or without treatment with EUK-134 (EUK). Data are presented as mean ± SEM for four to seven muscles in each group. *P < 0.05, **P < 0.01 versus CNT, ^##^ *P* < 0.01 versus AIA

There was no significant difference in the absolute twitch force of the EDL muscle between the rats with AIA and control rats (0.38 ± 0.02 versus 0.37 ± 0.02 N (*n* = 6); *P* > 0.05) (Fig. 2a). In contrast, the rates of force development (dP/d*t*) and relaxation (–dP/d*t*) in twitches were decreased by 27 and 29 % in AIA EDL muscles, respectively (Fig. 2a, b). Absolute tetanic force was ~20 % lower in AIA EDL muscles than that in controls at stimulation frequencies from 70 to 120 Hz (Fig. 2c). Although the cross-sectional area of AIA EDL muscles was somewhat decreased, tetanic force per cross-sectional area (i.e., specific force) was ~15 % lower than in control muscles at 100 and 120 Hz (*P* < 0.05 and *P* < 0.01, respectively) (Fig. 2d). Moreover, there was a left-ward shift of the force-frequency relationship in AIA EDL muscles with the frequency giving 50 % of the maximum force being significantly lower than in control muscles (31.9 ± 1.7 versus 40.9 ± 1.6 Hz (*n* = 6); *P* < 0.05).

In addition, there was a marked reduction in the specific force in AIA soleus muscles at stimulation frequency from 10 to 120 Hz (ranging from −33 to −42 % (*n* = 6–7); *P* < 0.01). Moreover, twitches were slower in AIA soleus muscles with dP/d*t* and –dP/d*t* being decreased by 27 and 34 %, respectively (*n* = 5–7; *P* < 0.05). No further experiments were performed on the soleus muscles. Thus, decreased tetanic force production and slowed twitch contractions are observed in both fast-twitch and slow-twitch muscles of AIA rats, which is consistent with previous results from CIA mice [5, 6].

We and others have proposed redox protein modification as one of the mechanisms underlying the intrinsic contractile dysfunction of the skeletal muscle in inflammatory conditions [5, 6, 21]. Therefore, we studied whether antioxidant treatment could prevent the arthritis-induced muscle weakness. AIA rats were then treated with the SOD/catalase mimetic EUK-134, which was injected intraperitoneally once daily starting the day after induction of AIA. EUK-134 treatment had no obvious effect on the development of arthritis, and the increase in the maximum diameter of the knee joint was similar in EUK-134-treated and PBS-injected control AIA rats (Fig. 2e). Nevertheless, the decrease in specific tetanic force was prevented in the EUK-134-treated AIA rats (Fig. 2f).

### Antioxidant inhibits redox modifications of actin in muscles from AIA rat

**Fig. 3:**
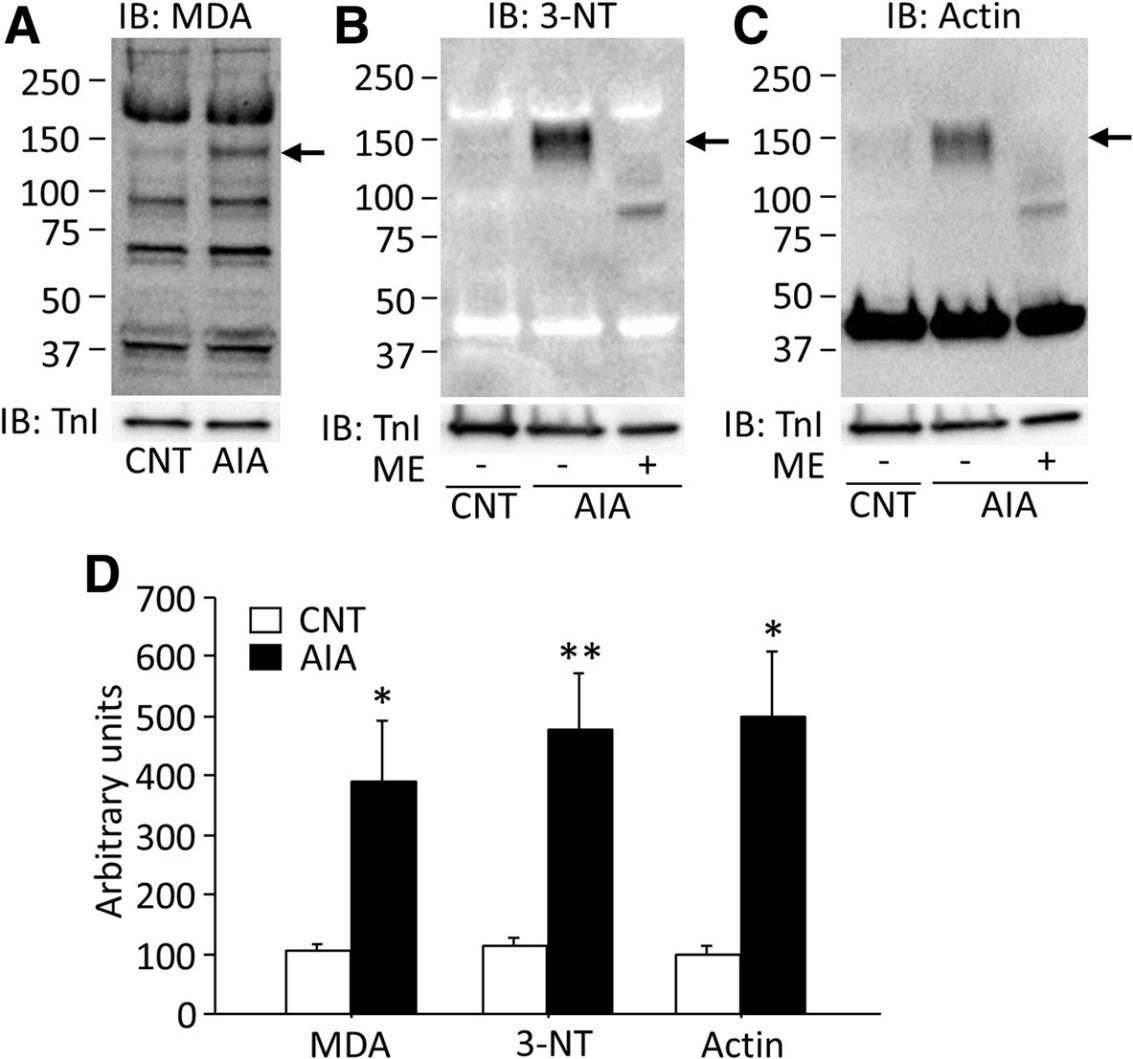
Malondialdehyde-protein adducts and 3-nitrotyrosine content are increased in actin aggregates from AIA EDL muscles. Representative Western blots for malondialdehyde (MDA)-protein adducts (**a**), 3-nitrotyrosine (3-NT) (**b**), and actin (**c**) in control (CNT) and AIA EDL muscles. In the 3-NT and actin blots, samples from AIA rats were run in the absence and presence of 2-mercaptoethanol (ME). **d** Intensities for the protein band at ~150 kDa (indicated by the *arrows*) in MDA-protein adducts, 3-NT, and actin were normalized to the troponin I (TnI) content. Data are presented as mean ± SEM for six muscles in each group. *P < 0.05, **P < 0.01 versus CNT

Due to previous results [5, 6, 21] and the present result that antioxidant treatment prevented muscle weakness in AIA rats, we next investigated whether the contractile defects were accompanied by signs of changed redox status in the AIA EDL muscle. MDA is a highly reactive by-product of lipid peroxidation. Western blotting showed several positive bands for MDA-protein adducts in both control and AIA muscles. Among these bands, EDL muscles from the rats with AIA showed a specific increase in MDA-protein adducts at ~150 kDa (Fig. 3a, d). 3-NT is considered to be a major product of peroxynitrite-derived radical interaction with tyrosine residues in proteins [9]. There was also a marked increase in 3-NT protein content at ~150 kDa in AIA muscles (Fig. 3b, d). It has previously been demonstrated that oxidative stress can induce the formation of actin aggregates [22]. In addition to the normal actin band seen at ~40 kDa, Western blots for actin showed a strong band at ~150 kDa in AIA muscles (Fig. 3c, d). It should be noted that the appearance of the ~150 kDa band in AIA muscles was not accompanied by any detectable decrease in the major ~40 kDa actin band, which shows that normally sized actin was still dominating. Intriguingly, the ~150 kDa bands for 3-NT and actin in AIA muscles disappeared when Western blotting was performed in the presence of the 2-mercaptoethanol (ME), which reduces disulfide bonds (Fig. 3b, c). Taken together, these results indicate the formation of ~150 kDa MDA- and 3-NT-containing and disulfide bond-dependent actin aggregates in AIA muscles.

**Fig. 4:**
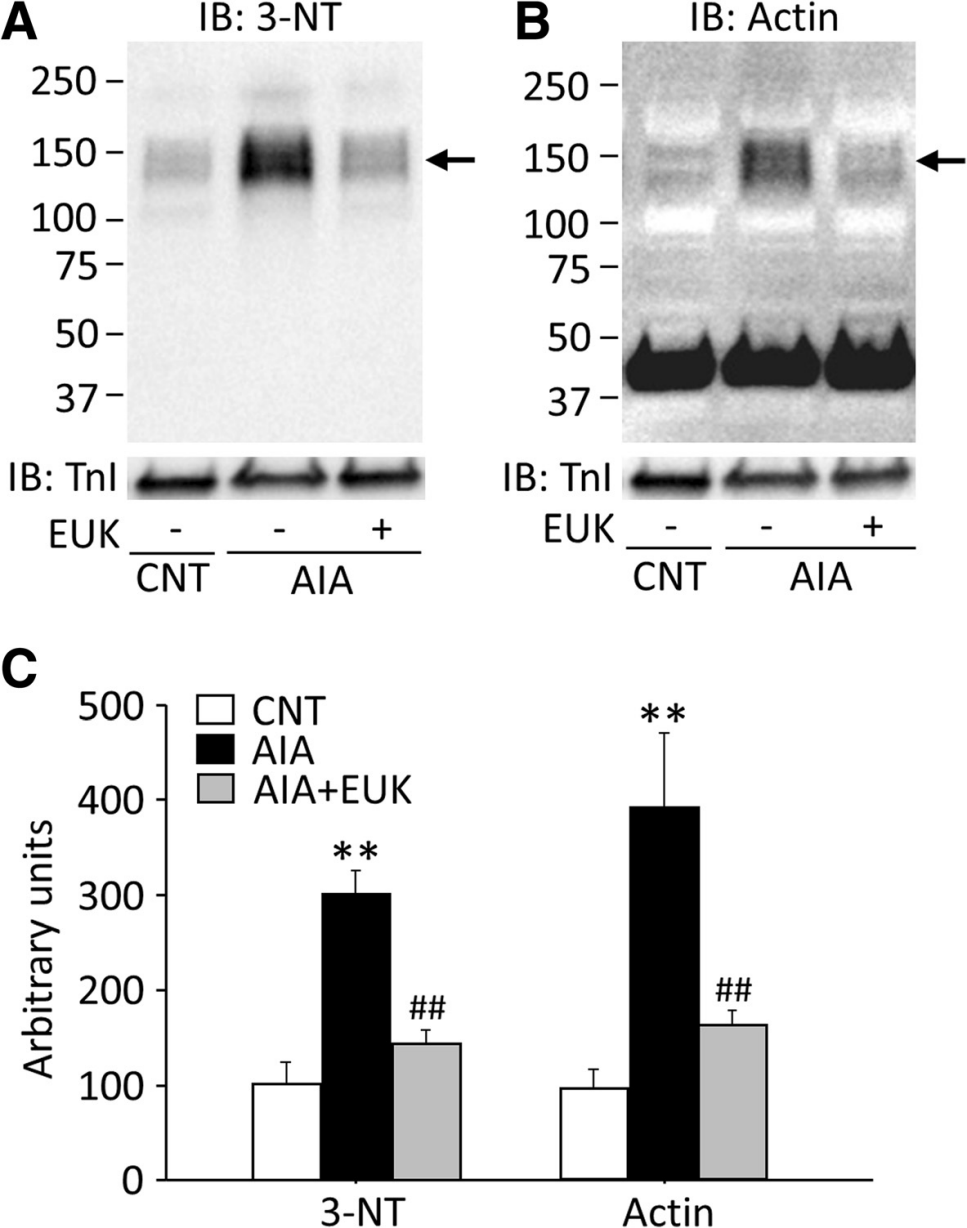
Antioxidant treatment of AIA rats prevents redox modifications of actin in EDL muscles. Representative Western blots for 3-nitrotyrosine (3-NT) (**a**) and actin (**b**) in EDL muscles from control (CNT) and AIA rats with or without treatment with EUK-134 (EUK). **c** Intensities for the protein band at ~150 kDa (indicated by *arrows*) in 3-NT and actin were normalized to the troponin I (TnI) content. Data are presented as mean ± SEM for four muscles in each group. **P < 0.01 versus CNT, ^##^ *P* < 0.01 versus AIA

The contractile deficiency was prevented when AIA rats were treated with the antioxidant EUK-134 (see Fig. 2f) and AIA muscles displayed major redox-induced actin modifications. Thus, we studied the effect of EUK-134 treatment on actin aggregates in EDL muscles of AIA rats. Importantly, the results show that the increase in 3-NT formation and aggregation of actin in AIA EDL muscles was prevented by EUK-134 treatment (Fig. 4a–c).

### Increased amount of redox-related proteins and inflammatory mediators in muscles from AIA rats

**Fig. 5:**
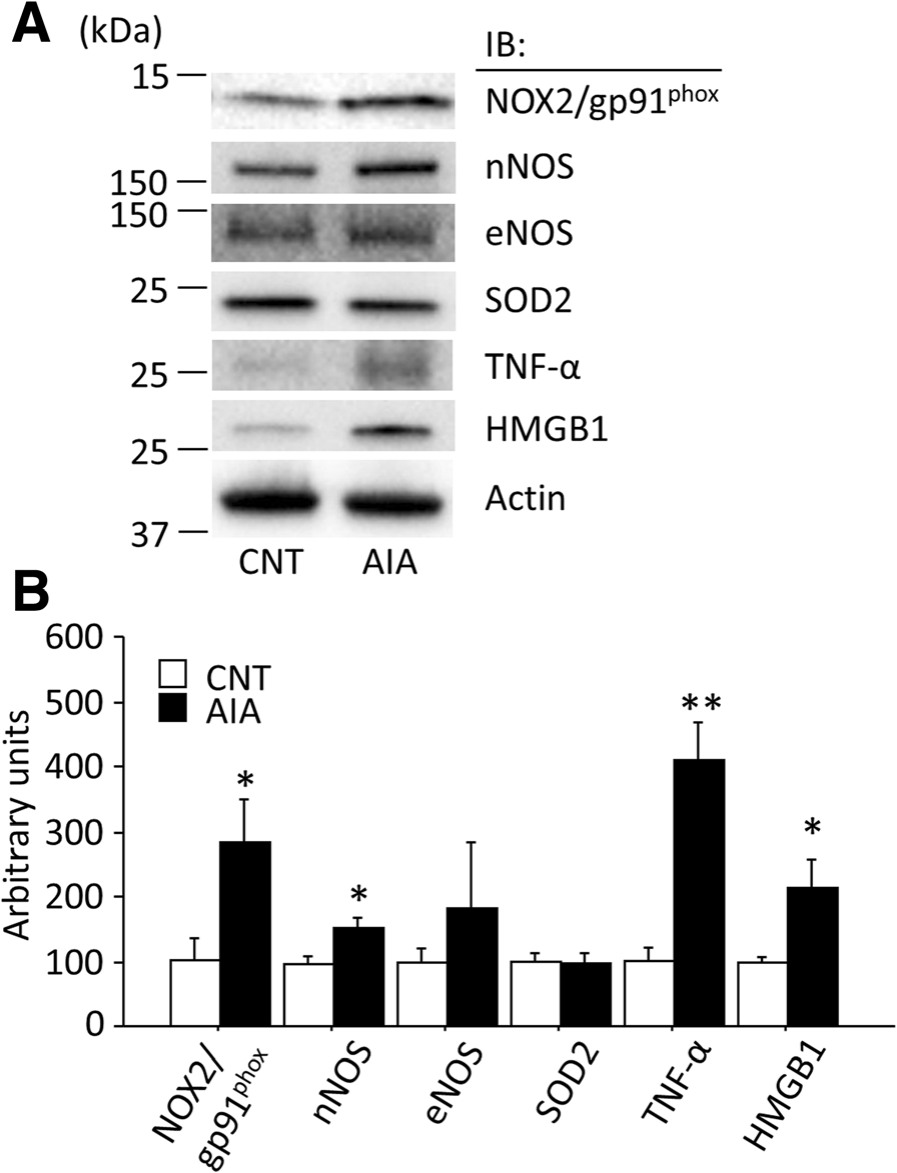
Expressions of redox-related proteins and inflammatory mediators are altered in AIA EDL muscles. **a** Representative Western blots illustrating the levels of NADPH oxidase (NOX2/gp91^phox^), neuronal nitric oxide synthase (nNOS), endothelial NOS (eNOS), superoxide dismutase 2 (SOD2), tumor necrosis factor α (TNF-α), and high-mobility group box 1 (HMGB1) in control (CNT) and AIA EDL muscles. The inducible NOS was not detected in either group. **b** Quantification of the levels of redox-related proteins normalized to actin content. Data are presented as mean ± SEM for three to six muscles in each group. *P < 0.05, **P < 0.01 versus CNT

ONOO^−^ is formed by the rapid, diffusion-controlled reaction of NO and superoxide. To assess the molecular mechanism of the accelerated production of ONOO^−^ in the AIA muscle, as indicated by the increased ~150 kDa 3-NT band, the expression of redox-related proteins were analyzed. The expressions of NOX2/gp91^phox^ and nNOS, but not eNOS, were significantly increased in AIA EDL muscles (Fig. 5a, b). iNOS was not detected in either group, and the expression levels of SOD2 were similar in the two groups.

We also measured the protein expression of the inflammatory mediators TNF-α and HMGB1, both of which have been suggested to induce redox stress [3, 23]. The protein expressions of both TNF-α and HMGB1 were substantially higher in AIA than in control EDL muscles (Fig. 5a, b).

## Discussion

We found a reduction in specific force production and slowed contractions in fast-twitch as well as slow-twitch muscles from the rats with AIA, which is consistent with our previous studies in CIA mice [5, 6]. Thus, in addition to muscle atrophy, impaired intrinsic contractility is a major cause of weakness in skeletal muscles of animal models of RA as well as in patients with RA [4]. Importantly, we here show that the decrease in force production was prevented when AIA rats were treated with the antioxidant EUK-134.

TNF-α-induced weakness has been shown to be mediated by an increase in muscle-derived oxidants, including NO derivatives [3]. Accordingly, brief exposure to TNF-α increased NO production and depressed specific force, and nNOS has been identified as the source of TNF-α-stimulated NO production in the skeletal muscle from guinea pig [24] and mouse [25], respectively. In agreement, we observed decreased specific force accompanied by increased levels of TNF-α and nNOS expression in AIA EDL muscles. Conversely, iNOS was not detected in AIA muscles in the present study. TNF-α, in combination with interferon (IFN)-γ, stimulates iNOS expression in C2C12 myocytes [26]. However, the expression of iNOS was increased directly after endotoxine injection and thereafter returned to baseline within ~24 h [27]. Our experiments were performed 3 weeks after induction of inflammation; hence, our data suggest that nNOS is the most important NOS isoform for the TNF-α-induced impaired contractility in chronic inflammatory conditions.

In addition to the NO derivatives, previous studies show that TNF-α stimulates superoxide production in the skeletal muscle via the mitochondrial electron transport chain [28] and phospholipase A_2_ [24]. TNF-α is also known to stimulate NADPH oxidases in human rheumatoid synovial tissue [29], which are enzymes that reduce molecular oxygen to superoxide [30], and we here show increased NOX2/gp91^phox^ protein expression in AIA muscles. Increased HMGB1 protein expression has been observed in the rat diaphragm with peritonitis, which was accompanied by signs of severe oxidative stress and contractile dysfunction [23]. We have previously shown that the stimulation with IFN-γ results in increased expression of HMGB1 in skeletal muscle fibers [31]. Thus, the upregulation of TNF-α and HMGB1 protein expression observed in the present AIA muscles is likely to cause increased superoxide production.

An intriguing finding of this study is the redox-dependent actin aggregates in AIA EDL muscles, which are most likely formed by disulfide bonds since they were reversed by the reducing agent 2-ME (see Fig. 3). The high amount of 3-NT, a product of tyrosine nitration mediated by ONOO^−^, in aggregated actin indicates a coordinated increase in NO and superoxide production in AIA muscles. The present results fit with a model where increased production of nNOS-derived NO and NOX2/gp91^phox^-derived superoxide favors ONOO^−^ production. Alternatively, nNOS is known to produce both NO and superoxide and may directly increase ONOO^−^ production [32]. Using a rat model of acute oxidative stress in the skeletal muscle, Fedorova et al. [22] detected actin-containing protein aggregates above a molecular mass of 100 kDa. Cys272 of the actin molecule is easily oxidized to sulfenic acid, slowly forming an intramolecular disulfide bond with Cys374, which alters the structure and exposes Cys17 to forming intermolecular disulfides [33]. Subsequently, Fedorova et al. [34] showed that carbonylation of actin results in significant reduction in actomyosin ATPase activity. Consistent with this, we observed a strong MDA band, which has been associated with increased protein carbonylation [35], at the same size (~150 kDa) as the actin aggregates. Furthermore, it has been shown that treatment of fibrillary (F)-actin with peroxynitrite donor produces a dose-dependent F-actin depolymerization and eventually impairs monomeric/F-actin equilibrium, leading to shortening of the actin filaments to a size smaller than that needed to activate myosin S1-ATPase [11]. Thus, these data suggest that the decreased actomyosin ATPase activity in AIA muscles is likely to be caused by redox modifications of actin.

Mn-Salen compounds, including EUK-134, have been proposed to possess distinct advantages over nonspecific antioxidant scavengers, such as combined SOD/catalase mimetic functions [12] and high translational value as the EUK series is developed for oral administration [36]. In addition to counteracting oxidative stress, Mn-Salen catalyzes the removal of ONOO^−^ and ameliorates nitrosative stress [13]. In a previous report, administration of EUK-134 reduced markers of oxidative stress and partially rescued a reduction in specific force production in the diaphragm from mdx mice [15]. We report here that daily intraperitoneal injection of EUK-134 prevented the AIA-induced 3-NT formation, aggregation of actin, and force depression. These results strongly support our notion that the oxidative/nitrosative modifications of actin result in contractile dysfunction in AIA muscles.

Previous studies provide evidence for a role of redox stress in the pathogenesis of RA [37]. Thus, we expected that treatment with EUK-134 would prevent the development of both arthritis and muscle weakness. However, the increase in knee diameter in AIA rats was not prevented by EUK-134 treatment (see Fig. 2e). The reason for this is unclear but could be due to the tissue-dependent differences in the levels of oxidative stress and/or concentration of EUK-134. AIA was induced in the knees by direct intraarticular injection of Freund’s complete adjuvant, and the oxidative stress might then be higher in these joints than in adjacent skeletal muscles. In contrast, EUK-134 was not specifically targeted to the afflicted joints since it was administered by intraperitoneal injections. Thus, it is conceivable that the concentration of EUK-134 in the knee joints did not reach high enough levels to prevent the development of arthritis.

In addition to decreased actomyosin ATPase activity, we also show decreased SERCA activity in the AIA muscle, which was not accompanied by any change in SERCA protein expression. This finding is consistent with the previous work by Strosova et al. [38] showing a redox-induced decrease in SERCA activity in the AIA skeletal muscle, which was not accompanied by decreased SERCA protein expression. Moreover, exposure to ONOO^−^ has been shown to depress SERCA activity [39]. Thus, increased ONOO^−^ production may have a central role in the decrease of both actomyosin ATPase and SERCA activities in AIA muscles. These alterations may account for the compromised contractility of AIA muscles; for instance, a decreased rate of cross-bridge cycling and SR Ca^2+^ uptake may directly explain the slowed twitch kinetics and a left-ward shift of force-frequency relationship in AIA muscles (see Fig. 2a–d).

## Conclusions

Using AIA rats, we show that arthritis can cause intrinsic contractile dysfunction in skeletal muscles. This dysfunction is likely to be induced by increased levels of the pro-inflammatory mediators TNF-α and HMGB1, which result in increased NO and superoxide production. This then leads to the formation of actin aggregates and decreased actomyosin ATPase activity and to impaired SERCA function. These deleterious changes were prevented by treating AIA rats with the antioxidant EUK-134, which then provides a tentative treatment of muscle weakness in patients with arthritis or other inflammatory diseases.

## Abbreviations

3-NT: 3-nitrotyrosine
AIA: adjuvant-induced arthritis
CIA: collagen-induced arthritis
DHPR: dihydropyridine receptor
EDL: extensor digitorum longus
eNOS: endothelial nitric oxide synthase
HMGB1: high-mobility group box 1
IFN: interferon
iNOS: inducible nitric oxide synthase
MDA: malondialdehyde
ME: mercaptoethanol
Mn-Salen: manganese-salen
MyHC: myosin heavy chain
nNOS: neuronal nitric oxide synthase
NO: nitric oxide
NOX2/gp91^phox^: NADPH oxidase 2 catalytic subunit gp91^phox^
ONOO^−^: peroxynitrite anion
RA: rheumatoid arthritis
RyR: ryanodine receptor
SERCA: sarcoplasmic reticulum Ca^2+^-ATPase
SOD2: manganese superoxide dismutase
TNF-α: tumor necrosis factor α

## References

[1] Häkkinen A, Kautiainen H, Hannonen P, Ylinen J, Makinen H, Sokka T (2006). Muscle strength, pain, and disease activity explain individual subdimensions of the Health Assessment Questionnaire disability index, especially in women with rheumatoid arthritis. Ann Rheum Dis.

[2] Stenström CH, Minor MA (2003). Evidence for the benefit of aerobic and strengthening exercise in rheumatoid arthritis. Arthritis Rheum.

[3] Reid MB, Moylan JS (2011). Beyond atrophy: redox mechanisms of muscle dysfunction in chronic inflammatory disease. J Physiol.

[4] Helliwell PS, Jackson S (1994). Relationship between weakness and muscle wasting in rheumatoid arthritis. Ann Rheum Dis.

[5] Yamada T, Place N, Kosterina N, Östberg T, Zhang SJ, Grundtman C (2009). Impaired myofibrillar function in the soleus muscle of mice with collagen-induced arthritis. Arthritis Rheum.

[6] Yamada T, Fedotovskaya O, Cheng AJ, Cornachione AS, Minozzo FC, Aulin C, et al. Nitrosative modifications of the Ca^2+^ release complex and actin underlie arthritis-induced muscle weakness. Ann Rheum Dis. 2014 (in press). 10.1136/annrheumdis-2013-205007 PMC4602262 24854355

[7] Fisher JS, Hasser EM, Brown M (1998). Effects of ovariectomy and hindlimb unloading on skeletal muscle. J Appl Physiol.

[8] Mercier C, Jobin J, Lepine C, Simard C (1999). Effects of hindlimb suspension on contractile properties of young and old rat muscles and the impact of electrical stimulation on the recovery process. Mech Ageing Dev.

[9] Szabó C, Ischiropoulos H, Radi R (2007). Peroxynitrite: biochemistry, pathophysiology and development of therapeutics. Nat Rev Drug Discov.

[10] Dutka TL, Mollica JP, Lamb GD (2011). Differential effects of peroxynitrite on contractile protein properties in fast- and slow-twitch skeletal muscle fibers of rat. J Appl Physiol.

[11] Tiago T, Ramos S, Aureliano M, Gutierrez-Merino C (2006). Peroxynitrite induces F-actin depolymerization and blockade of myosin ATPase stimulation. Biochem Biophys Res Commun.

[12] Baker K, Marcus CB, Huffman K, Kruk H, Malfroy B, Doctrow SR (1998). Synthetic combined superoxide dismutase/catalase mimetics are protective as a delayed treatment in a rat stroke model: a key role for reactive oxygen species in ischemic brain injury. J Pharmacol Exp Ther.

[13] Sharpe MA, Ollosson R, Stewart VC, Clark JB (2002). Oxidation of nitric oxide by oxomanganese-salen complexes: a new mechanism for cellular protection by superoxide dismutase/catalase mimetics. Biochem J.

[14] Kuwahara H, Horie T, Ishikawa S, Tsuda C, Kawakami S, Noda Y (2010). Oxidative stress in skeletal muscle causes severe disturbance of exercise activity without muscle atrophy. Free Radic Biol Med.

[15] Kim JH, Lawler JM (2012). Amplification of proinflammatory phenotype, damage, and weakness by oxidative stress in the diaphragm muscle of mdx mice. Free Radic Biol Med.

[16] Lawler JM, Kunst M, Hord JM, Lee Y, Joshi K, Botchlett RE (2014). EUK-134 ameliorates nNOSmu translocation and skeletal muscle fiber atrophy during short-term mechanical unloading. Am J Physiol Regul Integr Comp Physiol.

[17] Kanzaki K, Kuratani M, Mishima T, Matsunaga S, Yanaka N, Usui S (2010). The effects of eccentric contraction on myofibrillar proteins in rat skeletal muscle. Eur J Appl Physiol.

[18] Wada M, Okumoto T, Toro K, Masuda K, Fukubayashi T, Kikuchi K (1996). Expression of hybrid isomyosins in human skeletal muscle. Am J Physiol Cell Physiol.

[19] Bradford MM (1976). A rapid and sensitive method for the quantitation of microgram quantities of protein utilizing the principle of protein-dye binding. Anal Biochem.

[20] Simonides WS, van Hardeveld C (1990). An assay for sarcoplasmic reticulum Ca^2+^-ATPase activity in muscle homogenates. Anal Biochem.

[21] Supinski GS, Callahan LA (2007). Free radical-mediated skeletal muscle dysfunction in inflammatory conditions. J Appl Physiol.

[22] Fedorova M, Kuleva N, Hoffmann R (2009). Reversible and irreversible modifications of skeletal muscle proteins in a rat model of acute oxidative stress. Biochim Biophys Acta.

[23] Susa Y, Masuda Y, Imaizumi H, Namiki A (2009). Neutralization of receptor for advanced glycation end-products and high mobility group box-1 attenuates septic diaphragm dysfunction in rats with peritonitis. Crit Care Med.

[24] Alloatti G, Penna C, Mariano F, Camussi G (2000). Role of NO and PAF in the impairment of skeletal muscle contractility induced by TNF-alpha. Am J Physiol Regul Integr Comp Physiol.

[25] Stasko SA, Hardin BJ, Smith JD, Moylan JS, Reid MB (2013). TNF signals via neuronal-type nitric oxide synthase and reactive oxygen species to depress specific force of skeletal muscle. J Appl Physiol.

[26] Williams G, Brown T, Becker L, Prager M, Giroir BP (1994). Cytokine-induced expression of nitric oxide synthase in C2C12 skeletal muscle myocytes. Am J Physiol.

[27] El-Dwairi Q, Comtois A, Guo Y, Hussain SN (1998). Endotoxin-induced skeletal muscle contractile dysfunction: contribution of nitric oxide synthases. Am J Physiol.

[28] Li YP, Atkins CM, Sweatt JD, Reid MB (1999). Mitochondria mediate tumor necrosis factor-alpha/NF-kappaB signaling in skeletal muscle myotubes. Antioxid Redox Signal.

[29] Chenevier-Gobeaux C, Lemarechal H, Bonnefont-Rousselot D, Poiraudeau S, Ekindjian OG, Borderie D (2006). Superoxide production and NADPH oxidase expression in human rheumatoid synovial cells: regulation by interleukin-1beta and tumour necrosis factor-alpha. Inflamm Res.

[30] Lambeth JD (2004). NOX enzymes and the biology of reactive oxygen. Nat Rev Immunol.

[31] Grundtman C, Bruton J, Yamada T, Östberg T, Pisetsky DS, Harris HE (2010). Effects of HMGB1 on in vitro responses of isolated muscle fibers and functional aspects in skeletal muscles of idiopathic inflammatory myopathies. Faseb J.

[32] Stuehr D, Pou S, Rosen GM (2001). Oxygen reduction by nitric-oxide synthases. J Biol Chem.

[33] Shartava A, Monteiro CA, Bencsath FA, Schneider K, Chait BT, Gussio R (1995). A posttranslational modification of beta-actin contributes to the slow dissociation of the spectrin-protein 4.1-actin complex of irreversibly sickled cells. J Cell Biol.

[34] Fedorova M, Kuleva N, Hoffmann R (2010). Identification of cysteine, methionine and tryptophan residues of actin oxidized in vivo during oxidative stress. J Proteome Res.

[35] Burcham PC, Kuhan YT (1996). Introduction of carbonyl groups into proteins by the lipid peroxidation product, malondialdehyde. Biochem Biophys Res Commun.

[36] Rosenthal RA, Huffman KD, Fisette LW, Damphousse CA, Callaway WB, Malfroy B (2009). Orally available Mn porphyrins with superoxide dismutase and catalase activities. J Biol Inorg Chem.

[37] Sandhu JK, Robertson S, Birnboim HC, Goldstein R (2003). Distribution of protein nitrotyrosine in synovial tissues of patients with rheumatoid arthritis and osteoarthritis. J Rheumatol.

[38] Strosova MK, Karlovska J, Zizkova P, Kwolek-Mirek M, Ponist S, Spickett CM (2011). Modulation of sarcoplasmic/endoplasmic reticulum Ca^2+^-ATPase activity and oxidative modification during the development of adjuvant arthritis. Arch Biochem Biophys.

[39] Gutierrez-Martin Y, Martin-Romero FJ, Inesta-Vaquera FA, Gutierrez-Merino C, Henao F (2004). Modulation of sarcoplasmic reticulum Ca^2+^-ATPase by chronic and acute exposure to peroxynitrite. Eur J Biochem.

